# Role of imaging in glaucoma diagnosis and follow-up

**DOI:** 10.4103/0301-4738.73696

**Published:** 2011-01

**Authors:** Gianmarco Vizzeri, Sara M Kjaergaard, Harsha L Rao, Linda M Zangwill

**Affiliations:** 1 Department of Ophthalmology, Hamilton Glaucoma Center, University of California, San Diego, La Jolla, CA, USA; 2 L V Prasad Eye Institute, Banjara Hills, Hyderabad, India

**Keywords:** Confocal scanning laser ophthalmoscopy, glaucomatous progression, optical coherence tomography, retinal nerve fiber layer, scanning laser polarimetry

## Abstract

The purpose of the review is to provide an update on the role of imaging devices in the diagnosis and follow-up of glaucoma with an emphasis on techniques for detecting glaucomatous progression and the newer spectral domain optical coherence tomography instruments. Imaging instruments provide objective quantitative measures of the optic disc and the retinal nerve fiber layer and are increasingly utilized in clinical practice. This review will summarize the recent enhancements in confocal scanning laser ophthalmoscopy, scanning laser polarimetry, and optical coherence tomography with an emphasis on how to utilize these techniques to manage glaucoma patients and highlight the strengths and limitations of each technology. In addition, this review will briefly describe the sophisticated data analysis strategies that are now available to detect glaucomatous change overtime.

The detection of glaucomatous structural damage and change is one of the most important yet challenging aspects of glaucoma management. In recent years, imaging instruments, providing objective quantitative measures of neuroretinal rim thinning, retinal nerve fiber layer (RNFL) atrophy and excavation of the optic cup, are increasingly utilized in the clinical management of glaucoma patients. This is due in part to the provision of summary information that can be easily used in clinical management decisions. For example, most instruments now include a normative database with analyses indicating whether a measurement is “outside normal limits” or “within normal limits”. In addition, each provides a measure of image quality so that the clinician can determine whether the image is of sufficient quality to be utilized in clinical management decisions. With recent developments in technology such as spectral domain optical coherence tomography (SDOCT), the value of the imaging instruments in glaucoma management is likely to continue to grow.

Although *in vivo* imaging with confocal scanning laser ophthalmoscopy (CSLO), scanning laser polarimetry (SLP) and time-domain optical coherence tomography (TDOCT) has been commercially available for the management of glaucoma for over 10 years, interpretation of instrument results for detection of glaucoma and monitoring its progression remains a challenge. Only relatively recently, sophisticated data analysis strategies that efficiently analyze the high-dimensional retinal data have been developed and evaluated to detect glaucomatous change overtime.[1–9]

This review provides a brief update to recent reviews[10–17] describing advances in optical imaging for glaucoma management, with an emphasis on techniques for detecting glaucomatous progression and the newer SDOCT instruments.

## Confocal Scanning Laser Ophthalmoscopy

CSLO has been available for glaucoma detection since 1992. In brief, CSLO utilizes confocal optics to obtain multiple measures of retinal height at consecutive focal planes to provide a topographic map extending from the lamina cribrosa to the retinal anterior surface.

The latest generation CSLO, the Heidelberg retina tomograph III (HRT III) (Heidelberg Engineering, Heidelberg, Germany) employs the same image acquisition technology and similar software as the original Heidelberg retina tomograph classic (HRT), and the newer Heidelberg retina tomograph II (HRT II). Regardless of which instrument was used to acquire the images, all images can be analyzed with the new software (version 3.0 or higher) and imported into the newer instruments, although it remains to be evaluated whether progression analysis results are completely compatible when combining images acquired with the newer and older versions of the HRT. The instrument provides numerous stereometric parameters, including disc area, rim area, and cup area, to assist clinicians in assessing the anatomical features of the optic disc.

Numerous studies have shown that the reproducibility for the HRT and the HRT II stereometric parameters is good, with variability usually somewhat higher in glaucomatous eyes than in healthy eyes.[14,18–22] In addition, classification indices such as the Moorfields regression analysis (MRA) and the glaucoma probability score (GPS), which highlight regions as “outside normal limits” are among the HRT tools currently used to discriminate between healthy and glaucomatous discs.

## HRT Printouts

**Figure 1:**
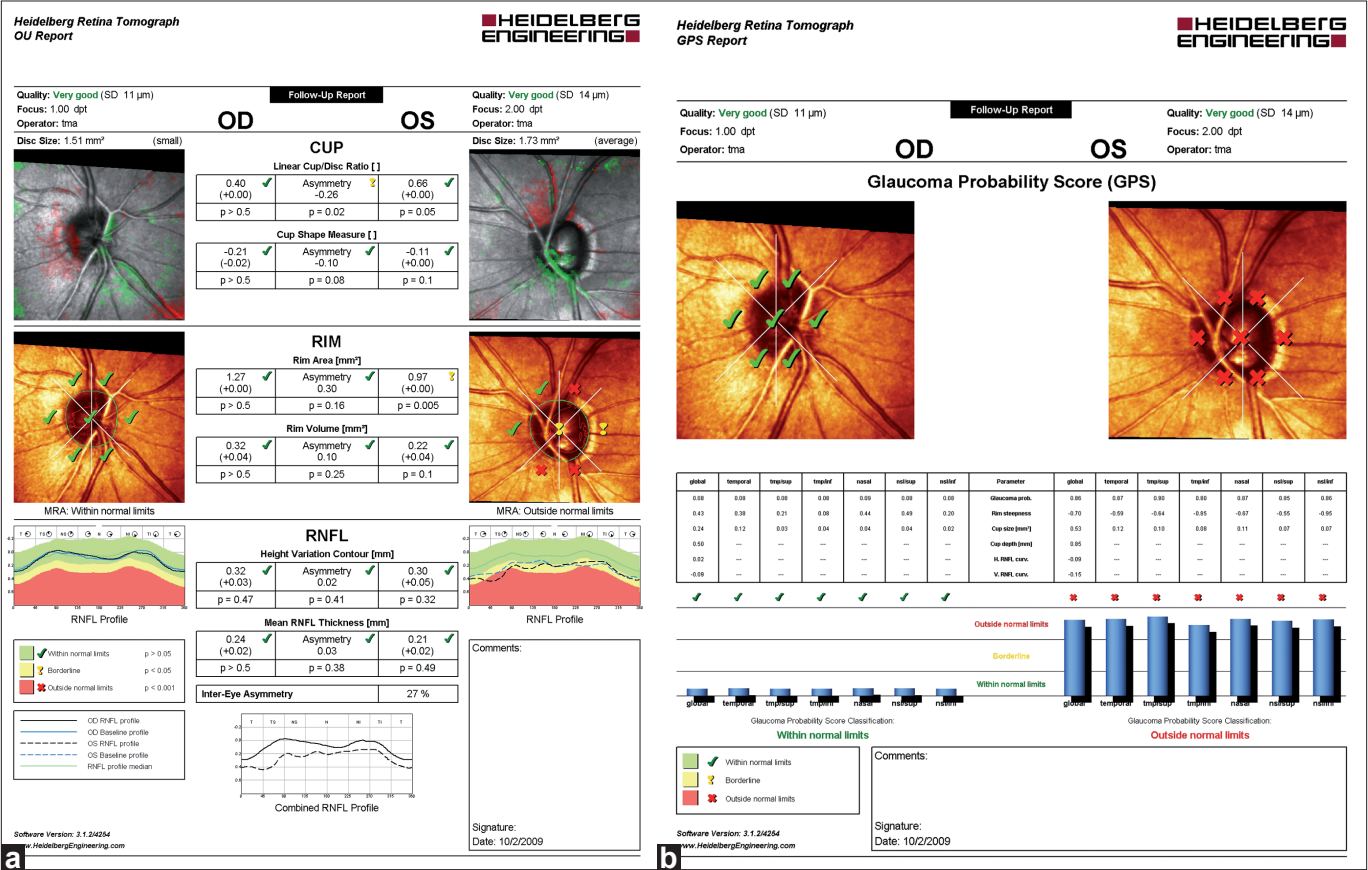
(a, b) HRT “Follow-up Report” (a) with results from the topographic change analysis (top) and the Moorfields Regression Analysis (middle). RNFL thickness measurements and inter-eye asymmetry are provided in the bottom section of the printout. HRT Glaucoma Probability Score (GPS) printout (b). The GPS classification is provided, superimposed on the optic disc image (top). Results from comparison with the internal normative database also are displayed in blue bars below. Abbreviation: RNFL: retinal nerve fiber layer

In Fig. 1a, a typical HRT “Follow-up Report” is divided into three parts to summarize cup, rim, and RNFL measurements for each eye, and asymmetry between eyes. Green checks indicate that the measurements are “within normal limits”, yellow exclamation points indicate measures are “borderline”, and red “x”s indicate measurements “outside normal limits”. All stereometric parameters require a user-drawn contour line to set a reference plane. Details of each section of the printout are provided below.

Information on image quality is reported at the top of the HRT Follow-up printout as color-coded text with the standard deviation (SD) in parentheses. In Fig. 1a, the quality of both right eye (OD) and left eye (OS) scans are “very good” (indicated in green at the top of the page), with SDs of 11 μm and 14 μm, respectively. Standard deviations of greater than 50 μm are considered as “poor quality” topographies, and the values are outlined in red to denote that the results should not be used or at least should be interpreted with caution.

The stereometric measurements of the cup are presented in the first section. In this example, linear cup/disc ratio and cup shape measurements of both eyes are within normal range, with symmetry of the linear cup/disc ratio indicated as “borderline”. The HRT images presented next to the cup summarize visually the topographic change analysis when there are sufficient follow-up scans available to complete the analysis (See “Topographic Change Analysis” in the “Detection of Glaucomatous Progression” section for additional details). In brief, pseudo-colors are used to indicate areas that are significantly elevated and areas that are significantly depressed (green and red colors, respectively) on consecutive follow-up examinations compared to baseline topographies.

In the center of the printout, overall neuroretinal rim area and rim volume measurements are presented and compared to the normative database. In this example, with the exception of OS rim area, which is “borderline”, the OD, OS, and asymmetry measurements are “within normal limits” (green checks). The HRT optic nerve head images presented next to the rim measurements visually summarize the results of the MRA which divides the ONH into six areas and compares rim area measurements of the examination to regression analysis results of rim area in normal eyes after adjusting for disc size and age. For the right eye of this example, the MRA is “within normal limits” overall (green checks in the middle of the image) and in each sector. In contrast, several sectors of the disc of the left eye are “outside normal limits”, as indicated by the red “x”s. Moreover, the MRA result is labeled with text “outside normal limits” as at least one sector is “outside normal limits”.

The bottom section of the printout shows the RNFL height variation contour and mean RNFL thickness values and the degree of asymmetry all “within normal limits”. The RNFL profile graphs on either side of the RNFL measurements map the RNFL measures along the optic disc margin of the baseline and current exam. In this example, the OS RNFL profile dips into the “outside normal limits” area in the temporal superior region.

## Glaucoma Probability Score

The GPS is a newer classifier that is based on a 3D geometric model with three parameters to characterize the optic disc (cup size, cup depth, and rim steepness) and two parameters to characterize the RNFL (horizontal RNFL curvature and vertical RNFL curvature). The parameters are then fed into a relevance vector machine classifier[23,24] that compares the results to a normative database, thus giving the probability of the disc being glaucomatous. The results are displayed on the printout as “within normal limits”, “borderline”, or “outside normal limits” both globally and for all six sectors.

In Fig. 1b, a typical GPS report is shown for the right and the left eyes. The printout is similar to the MRA printout described above. However, only the results from the relevance vector machine classifier, indicating whether the disc topography is “outside normal limits”, “borderline” and “within normal limits” are expressed, superimposed on the CSLO image. In the example provided, the OS sectoral and global GPS classifications are “outside normal limits”. The printout also provides results from several GPS parameters, such as cup size and rim steepness. Global and sectoral results from the GPS analysis can be further evaluated by looking at the height of the blue bars positioned at the bottom of the printout.

GPS and MRA have shown similar overall diagnostic accuracy, with the GPS tending to have higher sensitivity and lower specificity than the MRA.[25–28] Both classifiers are dependent on disc size, showing lower sensitivity and higher specificity in eyes with small discs compared to higher sensitivity and lower specificity in eyes with large discs.[26,27]

### Strengths and limitations

The strengths of the HRT include its large, race specific normative database, sophisticated analysis software for glaucoma detection and progression along with the ability to monitor quality control during image acquisition. In addition, the HRTII and HRTIII are theoretically backward compatible with the HRT classic instrument enabling continuity of topographic optic disc documentation of overtime. One of the limitations of HRT is that some topographic measurements are based on a reference plane constructed from a user-drawn contour line, so that operator input is required for particular analyses.[29] It should be noted that the topographic change analysis and GPS do not require a user-drawn contour line. Another limitation is that in some eyes, intraocular pressure (IOP) can significantly influence HRT measurements.[30]

## Scanning Laser Polarimetry

SLP takes advantage of the birefringence property of the RNFL that modifies the polarization of the light (retardation) when illuminated. The retardation is proportional to the thickness of the birefringent tissue, thus allowing the instrument to obtain objective and quantitative measurements of the RNFL thickness. The reliability of the measurements is dependent, at least in part, on the machine’s ability to extract the RNFL retardance from the total ocular retardance, since the cornea and the lens also exhibit some degree of birefringence.

The commercially available SLP instruments are the GDx VCC (variable cornea compensation) and the latest GDx ECC (enhanced corneal compensation) (both from Carl Zeiss Meditec, Dublin, CA, USA). The GDx has undergone numerous implementations over the years, with the goal of providing more reliable and reproducible measurements of the RNFL thickness. Initially, the instrument was equipped with a fixed corneal compensation. However, the device was not able to adjust for the variability of corneal thickness and properties among different individuals. This important issue was later addressed by providing the GDx with a variable corneal compensator. The GDx has been shown to discriminate well between glaucomatous and healthy eyes.[31] However, a major challenge in the ability of the instrument to describe the RNFL thickness pattern relies in the occurrence of atypical retardation patterns (ARPs), likely the result of poor signal-to-noise ratio (SNR) as a consequence of light scattering in the eye. ARP scans typically show irregular patches of elevated retardation values that do not match the expected retardation based on the RNFL anatomy. The measurements could either mask true RNFL loss or give a false glaucomatous appearance. Medeiros *et al*. showed that the appearance of ARPs had a significant negative influence on the ability to detect progressive RFNL loss with the GDx VCC.[32] The newer instrument, the GDx-ECC was designed to limit the occurrence of ARPs. Recent studies have shown that in general the ability to discriminate between glaucomatous and healthy eyes is higher with the ECC compared to previous SLP technology, particularly in eyes with earlier stages of disease and severe ARPs.[33,34]

## GDx Printout

**Figure 2:**
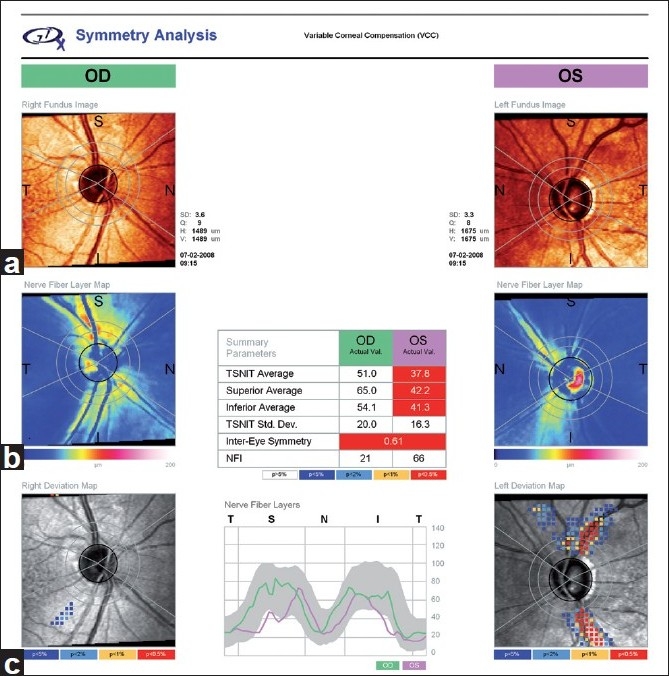
GDx VCC symmetry analysis printout displaying a fundus image of the optic nerve (a), the nerve fiber layer map in pseudo-colors (b), and the deviation map (c). VCC: variable corneal compensator

The GDx VCC symmetry analysis printout is divided into three sections [Fig. 2]. In this example, the scans are of good quality with “Q” values presented next to the “fundus image” for the right and left eyes being 9 and 8, respectively. The nerve fiber layer map is shown in pseudo-colors for the right and the left eyes (center), with brighter colors indicating a thicker RNFL. The deviation map compares RNFL thickness results to the instruments’ normative database (bottom). In the example provided, the OS RNFL is particularly thin and “outside normal limits” in the supero-temporal and infero-temporal regions, as evidenced by the red pattern in the left deviation map. The RNFL thickness pattern for the two eyes is visualized, and a symmetry analysis is provided at the bottom of the printout. Several important RNFL thickness parameters, such as the temporal superior nasal inferior temporal (TSNIT) average and the nerve fiber indicator (NFI), also are displayed with pseudo-colors used to flag parameters “borderline” (blue and yellow) or “outside normal limits” (red).

### Strengths and limitations

The major strength of the SLP relies in the ability to obtain reproducible measurements of the RNFL thickness without pupil dilation, a reference plane or magnification correction. Some of the limitations with previous versions of the device, such as the variable corneal birefringence or the occurrence of ARPs have been overcome with the introduction of the GDx VCC and the software enhanced GDx ECC, respectively. However, ARPs are still present even when using GDx ECC in some patients, and this must, therefore, be considered a limitation of the SLP technology. In addition, the newer GDx instruments are not backward compatible with older instruments, so that RNFL measures acquired with different GDx instruments are not comparable.

## Time Domain Optical Coherence Tomography

Optical coherence tomography (OCT) is an imaging method analogous to ultrasound B mode imaging, except that light instead of sound is used to acquire high-resolution images of ocular structures. By applying the principles of low coherence interferometry to light backscattered from ocular structures, OCT provides cross-sectional images of the macula, the peripapillary retina, and the optic nerve head. The final image is artificially color-coded by the OCT software. High reflective tissue, such as the RNFL, appears green and yellow whereas less reflective tissue has darker colors such as black and blue. TDOCT is the term now widely used to distinguish Stratus OCT from the newer SDOCT technology (see “Spectral Domain Optical Coherence Tomography” section below for more details). With TDOCT, the different echo time delays produced by the back reflected light are measured separately. The first TDOCT was introduced over a decade ago. The commercially available time domain Stratus OCT (Carl Zeiss Meditec, Dublin, CA, USA) provides better resolution (8-10 μm), increased number of A-scans, and reduced need for pupil dilation compared to previous OCT instruments.

Several studies have reported good reproducibility of RNFL thickness measurements using Stratus OCT in normal and glaucomatous eyes and good diagnostic ability for glaucoma detection.[35–40] In addition, studies performed using previous and current versions of the OCT have demonstrated its ability to detect RNFL thickness damage in glaucomatous eyes in agreement with red-free RNFL photographs and visual fields.[41–43]

## Stratus OCT Printout

**Figure 3:**
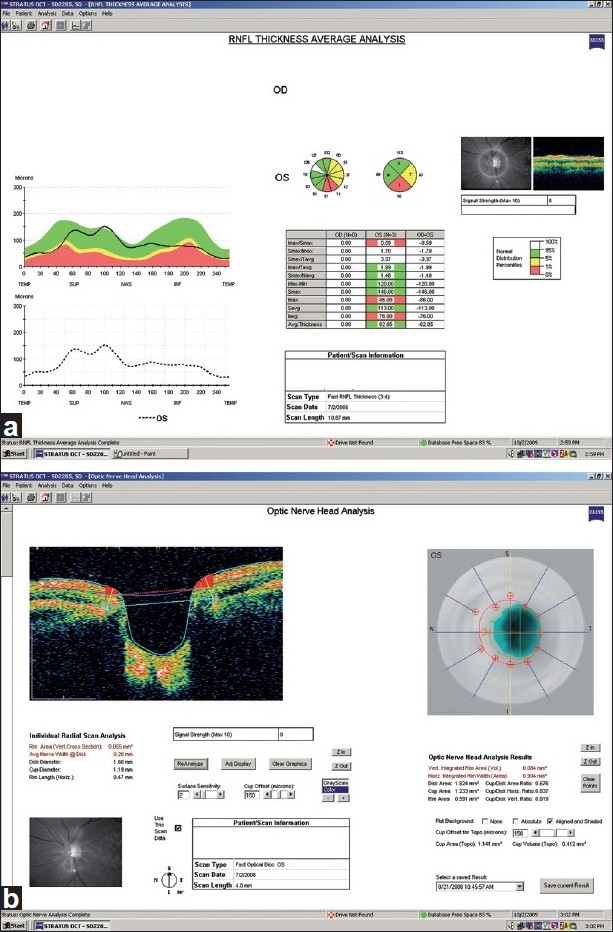
(a, b) Stratus OCT RNFL thickness average analysis (a) and optic nerve head analysis (b) of the left eye. RNFL: retinal nerve fiber layer

The Stratus OCT RNFL thickness average analysis is shown in Fig. 3a for OS. This analysis displays the RNFL thickness profile for the study eye (black line), superimposed on the characteristic double hump profile pattern resulted from the internal normative database. In this example, the RNFL is clearly thinner in the inferior region. RNFL thickness measurements by sectors and clock hours also are shown in the center, above several other calculated parameters. In the example shown, the inferior sector and corresponding clock hours are flagged as “red”, i.e., “outside normal limits”. For quality assessment, the average signal strength (from 1 to a maximum of 10) for the Fast RNFL thickness protocol is provided. For this scan, the signal strength is “8” indicating good quality.

The Stratus OCT optic nerve head analysis is shown in Fig. 3b for a good quality (signal strength = 8) OS scan. This analysis results from data processing derived from six radial scans centered on the optic disc by the operator. The instrument provides optic nerve head analysis results that include several optic disc parameters, such as disc area, cup area and rim area along with an image (above) that describes the contour of the disc (in red) and the area of the cup (in blue). Individual radial scan measures are also provided (top left). Each scan can be evaluated separately for quality purposes and to ensure that retinal structures are properly identified by the segmentation algorithm.

### Strengths and limitations

Strengths of the Stratus OCT include its ability to measure peripapillary RNFL thickness without the need for a reference plane or magnification correction, and that RNFL, optic disc, and macula scans are available in one instrument. There is little evidence, however, that combining OCT information from RNFL thickness, ONH topography, and macula measurements improves glaucoma diagnostic accuracy over each of the analyses alone.[38]

A limitation of the Stratus OCT relies in the fact that the instrument acquires a limited amount of data for each of its scanning protocols. For example, for the fast ONH scan, there is interpolation of data between the six radial scans. In addition, there is no scan registration available. Therefore, the instrument relies in an operator to consistently center the scan circle during each visit. A landmark feature is available to facilitate image acquisition at the same location at each visit. Recent studies have shown that scan circle misalignment can affect RNFL thickness measurements.[7,44,45] There also appears to be a positive linear relationship between signal strength and RNFL thickness (i.e., the greater the signal strength, the greater the RNFL thickness), and this should be taken into account when interpreting a single scan or a series of scans overtime.[7,46,47] Finally, the Stratus OCT is not backward compatible with previous OCT instruments.

## Spectral Domain Optical Coherence Tomography

Until recently, clinically available TDOCT instruments have used a technique to obtain images wherein the different echo time delays produced by the back reflected light were measured separately leading to slow acquisition time and limited data gathering. With the introduction of SDOCT, it has become possible to image ocular structures with better resolution and with a much faster scan rate. These instruments are known as “Spectral” or “Fourier domain” because echo time delays of light are measured by taking the Fourier transform of the interference spectrum of the light signal. Because OCT with Fourier domain detection can measure all light echoes from different delays simultaneously, it has a dramatic speed compared with TDOCT.[48–50] Compared to TDOCT which collects 400 axial measurements per second with an axial resolution of around 10 μm, the scan rate of SDOCT is at least 20,000 axial measurements per second with an axial resolution of 5 μm. Shorter image acquisition time leading to less eye motion artifacts, acquisition of large number of data points to allow three-dimensional imaging and scan registration from session to session, and higher resolution with more precise segmentation of retinal layers are some of the advantages of SDOCT over TDOCT.

There are several SDOCT devices commercially available at this point in time, each with several unique advantages. For example, the RTVue (Optovue Inc., Fremont, CA) offers the ganglion cell complex (GCC) protocol, which is designed to measure the inner retinal thickness to include the nerve fiber layer, ganglion cell layer, and the inner plexiform layer, collectively called the GCC, believed to be the primary region of affection in glaucoma. The Cirrus HD-OCT (Carl Zeiss Meditec Inc., Dublin, CA) includes the optic disk cube 200 × 200 protocol that provides automated alignment of the scan circle around the optic disc, allowing manual centering of the measurement cube on the optic disc center after image acquisition in cases of decentration due to eye movements. The Spectralis OCT (Heidelberg Engineering, Dossenheim, Germany) incorporates a real time eye tracking system that couples CSLO and SDOCT scanners to adjust for eye movements and to ensure that the same location of the retina is scanned over time. This method allows B-scans to be re-sampled to improve the SNR ratio. The Topcon 3D OCT-1000 (Topcon, Paramus, NJ) has the advantage of combining a nonmydriatic fundus camera with the imaging capabilities of SDOCT technology.

In general, all SDOCT devices incorporate sophisticated software for image acquisition and data analysis that provides real-time image quality information to the operator, and compares measurements to normative databases with color-coded results as red (“outside normal limits”), green (“within normal limits”), and yellow (“borderline”). It is important to note that these systems are evolving rapidly, and it is likely that numerous software enhancements will be made available in the near future.

**Figure 4:**
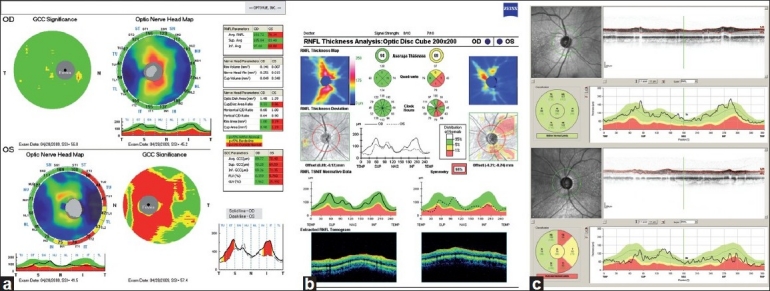
(a-c) Printouts from the commercially available spectral domain optical coherence tomography devices. (a) RTVue, (b) Cirrus HD-OCT, and (c) Spectralis OCT

Figs. 4a–c are printouts from the RTVue, Cirrus HD-OCT, and Spectralis OCT, respectively. RTVue printout [Fig. 4a] provides the color-coded GCC, and the optic nerve head maps with their respective parameters in a tabular form. Image quality is summarized as the “signal strength index” (SSI) with scans above 45 considered good quality. The optic nerve head protocol provides the optic nerve head parameters as well as the RNFL parameters similar to the Stratus OCT optic nerve head and RNFL maps. The GCC protocol, in addition to the inner retinal thickness at the macula, also provides two other parameters called global loss volume (GLV) and focal loss volume (FLV). GLV measures the average amount of GCC loss over the entire GCC map, and FLV measures the average amount of focal loss over the entire GCC map, much like the total and pattern deviation maps in the visual fields.

The Cirrus HD-OCT printout [Fig. 4b] is of the optic disc cube protocol, which is a three-dimensional scan of a 6 × 6 mm[2] area centered in the optic disc. Image quality is measured by signal strength with values six or above considered good quality. The printout provides summary information in several sections including the RNFL thickness map and RNFL thickness deviation (analogous to the GDx Nerve Fiber Layer and Deviation Maps), and RNFL TSNIT normative data (with presentation of information similar to that of the Stratus OCT).

The Spectralis OCT RNFL thickness printout provides the RNFL scan and profile corresponding to a circle of 3.4 mm diameter centered on the optic disc, as shown in Fig. 4c. The RNFL thickness around the optic disc is measured in six sectors corresponding to the sectors generated by the HRT MRA and GPS. A SNR of 15 dB or higher is considered good quality.

Studies have shown the SDOCT devices to have excellent intrasession repeatability for RNFL,[51–53] ONH, and macular measurements.[54–56] Leung *et al*. also found that the intervisit variability of Cirrus HD-OCT was significantly lower than that of Stratus OCT.[57]

There have been few studies to date evaluating the diagnostic performance of these devices. Leung *et al*.[57] evaluated RNFL measurements of Cirrus HD-OCT and found that the average (AUC, 0.962), superior (AUC, 0.963), and inferior (0.949) quadrant RNFL thickness measurements had the best discriminating ability to differentiate normal eyes from eyes with glaucomatous visual field defects. They also found that the diagnostic performance of Cirrus HD-OCT was similar to that of Stratus OCT.[57] Sehi *et al*. evaluated the RNFL measurements of RTVue, and they too found that the diagnostic ability of RTVue was similar to Stratus OCT; inferior (0.95), average (0.87), and superior (0.79) quadrant RNFL measurements had the best AUC in their study.[58] Sung *et al*. tested the sensitivity and specificity of the normative classification of Cirrus HD-OCT and found that the sensitivity (64%) and specificity (100%) of the average RNFL measurement of Cirrus HD-OCT was better than the sensitivity (40%) and specificity (96.7%) of the Stratus OCT.[59] There are no reports yet on the diagnostic performance of the other SDOCT devices as well as the ONH and the macular measurements of SDOCT devices.

Even though there appears to be no improvement in the diagnostic accuracy of the SDOCT over TDOCT in diagnosing glaucoma, there are still some potential benefits of the newer technology apart from faster scan acquisition time and improved resolution. The increased number of scans obtained by SDOCT may allow for the development of better registration algorithms which might have a superior performance in longitudinal RNFL assessment and for judging progression. This aspect will need to be evaluated in future studies.

### Detection of glaucomatous progression

Glaucoma is a slowly progressing optic neuropathy characterized by the loss of retinal ganglion cells and their axons. Therefore, the detection of glaucomatous progression is a critical aspect of glaucoma management. The identification of glaucomatous changes, such as progressive thinning of the RNFL, not only can help clinicians in confirming the initial diagnosis but, more importantly, can alert them that further treatment may be required to prevent visual impairment due to glaucoma.

Imaging instruments offer the advantage of providing large amount of reproducible data that can be used to develop analysis strategies for detecting change over time. Ideally, imaging instruments should be able to detect clinically relevant changes at the level of the disc or the RNFL that are greater than the variability of the measurements. With imaging instruments, multiple scans are obtained at each imaging session, so that measurement variability can be calculated, both globally and regionally. It is, therefore, possible to identify regions of the optic disc and RNFL that have changed significantly (greater than the variability of the measurements) overtime. In addition, because it is important to document that the change is repeatable, these instruments have the potential to automatically identify regions of the optic disc and RNFL that show significant and consistent change over several consecutive imaging sessions, therefore confirming that change has occurred.

Recent reports have suggested that imaging technologies have the potential to detect glaucomatous structural changes. Studies with HRT, GDx, and Stratus OCT, for example, have shown that on average the decrease in rim area or RNFL thickness occurs at a faster rate in eyes progressing overtime compared to non-progressing eyes, with the assessment of progression based on stereophotography or visual fields.[6,9,60,61]

However, for the method to be useful in clinical practice, it is important for change to be detected in eyes of individual patients. For this purpose, most imaging technologies now incorporate specific software that allows for clinicians to detect significant change in a single eye with adequate follow-up. Preliminary studies have shown that these methods are capable of detecting change in glaucomatous eyes or eyes of glaucoma suspects.[60,62]

It is important to note that agreement between various methods of analysis, such as progression by stereophotography or visual fields, and progression by imaging techniques is generally poor and further studies are needed to determine whether a longer follow-up will yield a better agreement between methods.[2,4,5,9] It will also be important to better characterize what constitutes a clinically significant change in glaucoma.

Below is a brief description of the methods used by HRT, GDx, and Stratus OCT for detecting glaucomatous progression.

**HRT Topographic Change Analysis:** The topographic change analysis (TCA) is currently the primary method for assessing glaucomatous change using the HRT.[3–5,8,63–65] By accounting for the effect of scan variability and location of topograph height measurements, TCA describes significant and repeatable changes in picture elements (so-called superpixels, i.e., 4 × 4 pixels) over the topographic map, with red demonstrating depression and green demonstrating elevation compared to baseline. TCA change summary parameters can be used to describe size and location of regions of change.

**Figure 5:**
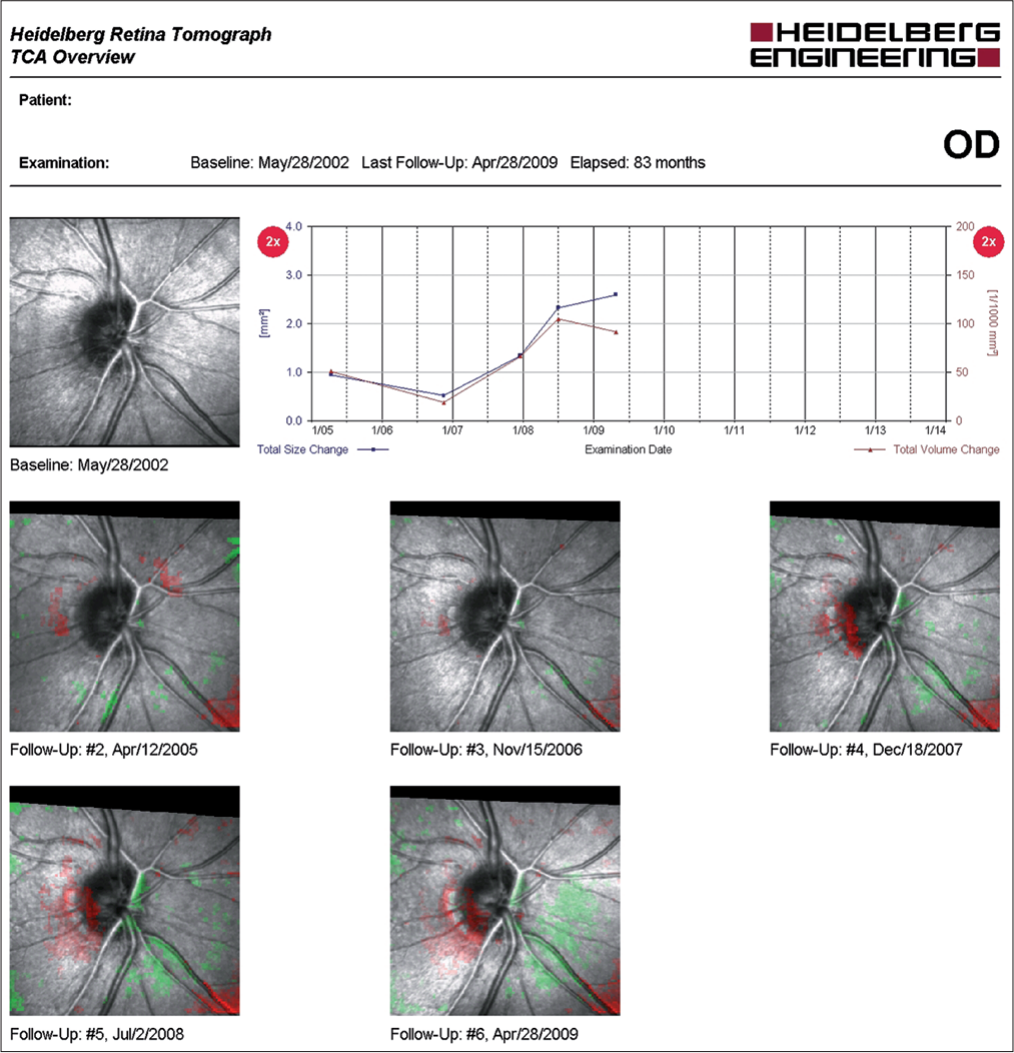
Topographic change analysis overview of the right eye. The presence of red pixels inside the disc at three consecutive follow-up exams is indicative of glaucomatous progression. In addition, the graph shows change overtime beginning in 2008 with an increase in the total size and volume change of the superpixel cluster

Fig. 5 shows an example of a TCA printout of an eye that has shown significant change over time, indicative of increased optic disc cupping and neuroretinal rim thinning. The change, indicated by the presence in the 2007 scan of red superpixels within the optic disc margins at the infero-temporal region, appears to occur inside the disc margins and by definition is repeatable in follow-up scans. In addition, the graph shows change overtime beginning in 2008 with an increase in the total size and volume change of the superpixel cluster. For convenience, TCA results are also presented in the HRT “Follow-up Report” [Fig. 1a].

**GDx-Guided Progression Analysis:** The GDx VCC-guided progression analysis (GPA) software evaluates and compares SLP images acquired during follow-up and labels progression as “Possible Progression“ (shown in yellow) if significant decrease in RNFL thickness is detected once, “Likely Progression” (shown in red) if significant reduction is detected in at least two consecutive exams, and “Possible Increase” (shown in purple) if an increase in RNFL thickness is detected. Two types of analysis are available, depending on whether one or three scans are obtained at each visit. The “fast mode” compares the two last images with the two baselines; change is identified as significant if it meets a predetermined criteria based on an independent group of eyes. In contrast, the “extended mode” uses a mean of three images at each visit, measures the variability of the mean image, and identifies significant change that is greater than the variability measured for that individual eye.

**Figure 6:**
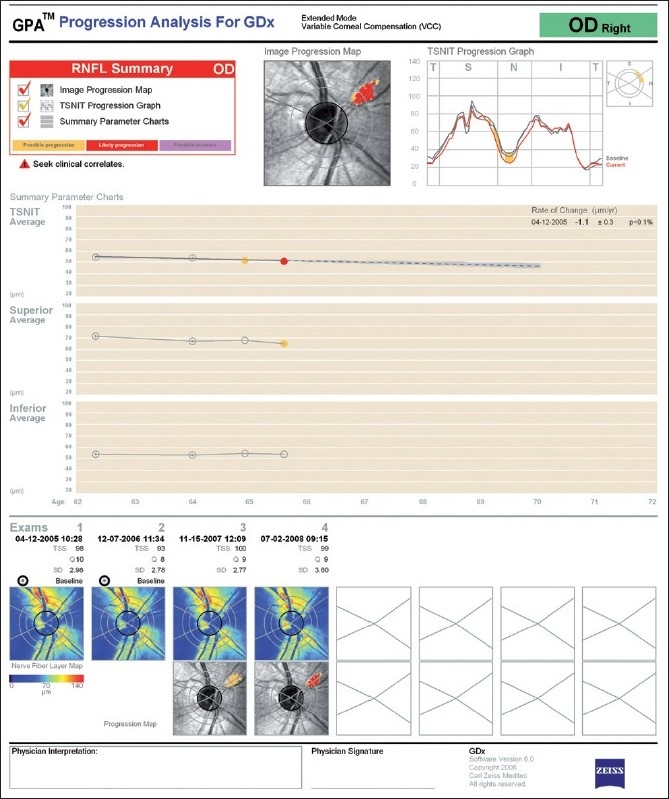
GPA progression analysis for GDx of the right eye. The image progression map shows a region of “Likely Progression” in the supero-nasal quadrant. The rate of change is shown below, and it is significant for the parameter “TSNIT Average”

An example of the GDx GPA extended mode analysis is shown in Fig. 6. The GDx GPA uses two different statistical analyses to determine significant change, and provides the results in three different maps, each focusing on a specific pattern of damage. The “Image Progression Map” represents a fundus image with color-coded areas, flags as significant a cluster of at least 150 adjacent pixels showing changes in RNFL measurements compared to the two individual baselines. In this example, possible change is identified as red pixels in the superior nasal region. According to the manufacturer, the image progression map was designed to be more sensitive to narrow, focal RNFL loss.

The “TSNIT Progression Graph” shows RNFL thickness measurements around the optic disc (TSNIT stands for the sectors around the optic disc, T for temporal, S for superior, N for nasal, and I for inferior) where the current exam (red line) is plotted over the baseline exams (gray lines). The GDx calculation circle is divided in 64 segments; and requires significant change in at least four adjacent segments to be flagged as progression. In this example, likely possible change is identified in the superior nasal regions.

The third-image-based map is the “Summary Parameters Charts”, in which three parameters are plotted: the TSNIT average, the superior average, and the inferior average. These parameters are displayed from all included images in chronological order, and a regression line is drawn if the last one shows “Likely Progression” and there is a significant linear trend (*P* < 5%). In these cases, the corresponding rate of change (given in μm/year with 95% confidence interval) and a *P* value is also provided. The “Summary Parameters Charts” was designed to be more sensitive to diffuse change. In the example provided, a significant change can be observed in the supero-nasal region in the Image Progression Map (flagged as “Likely Progression”) and a negative linear trend is also found (-1.1 ± 0.3 μm/year).

**Figure 7:**
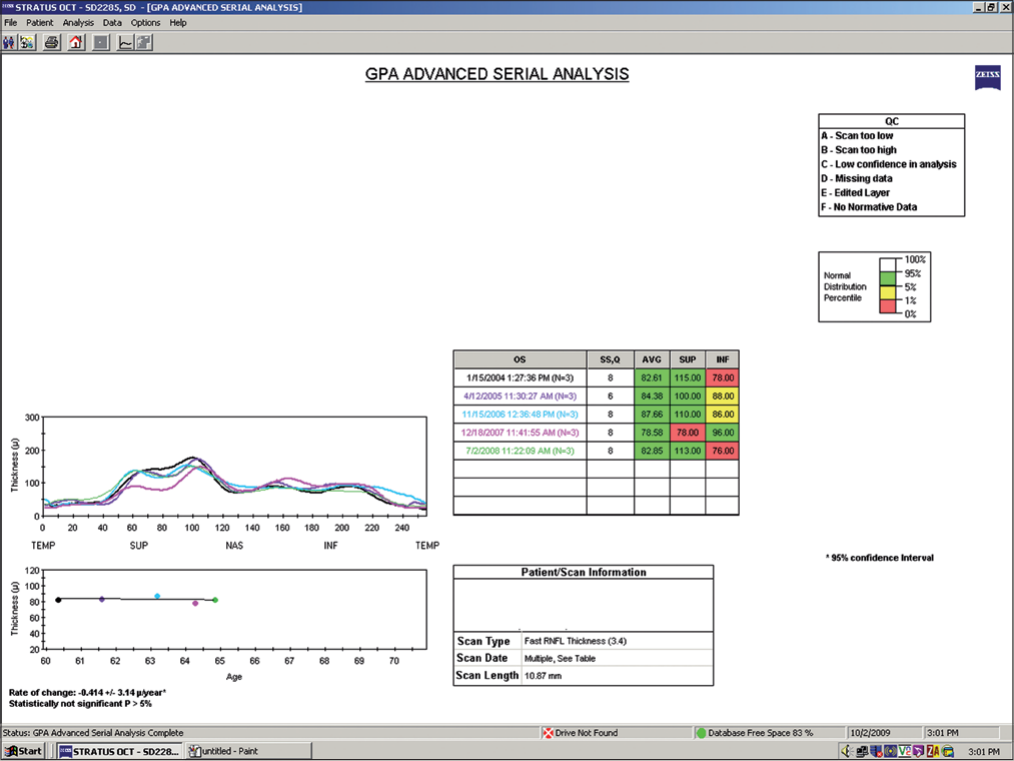
Stratus OCT GPA advanced serial analysis of the left eye. The rate of change of the average RNFL thickness, shown at the bottom left, is statistically not significant. Abbreviation: RNFL: retinal nerve fiber layer

**Stratus OCT-Guided Progression Analysis:** The commercially available Stratus OCT now includes Guided Progression Analysis (GPA) (software version 6.0) which evaluates and compares Stratus OCT scans acquired during follow-up and reports a summary analysis for progression in an individual eye after automated consideration of expected test-retest variability. An example of this type of analysis is shown in Fig. 7. All selected scan patterns are visualized in different colors in a double hump profile for visual comparison among scans (left). The corresponding rate of change (given in μm/year with 95% CI), and a *P*-value is provided. In the case shown, although a negative rate of change of -0.414 ± 3.1 μm/year was found, this was not statistically significant (*P* > 5%). The report also provides the signal strength values, a measure of image quality for each follow-up scan along with the average, superior, inferior RNFL thickness measurements.

## Conclusions

Imaging instruments show promise for improving the documentation and detection of optic disc and RNFL changes for clinical management of glaucoma. Each instrument is in a different stage of development with important software improvements anticipated, particularly for detecting change overtime. Sophisticated computer intensive techniques have been reported that show promise for improving detection of structural change overtime.[8,66,67] With increased computing capabilities now available, these and other techniques may become standard tools in imaging instruments in the near future.

It is important to remember that the quality of the scan and severity of glaucoma can influence the diagnostic accuracy of all imaging instrument results.[7,44,68] Predictably, the diagnostic accuracy of even the most sophisticated analyses of optic disc and RNFL data may be limited if poor quality scans are used, and will be much higher in eyes with advanced glaucoma than in eyes with early disease. It is, therefore, essential the clinicians understand the strengths and limitations of each instrument and interpret the data accordingly. Moreover, it is important to use good quality images in conjunction with a complete clinical examination and assessment of visual function for patient management decisions.
